# Supplementary material for: Multifunctional CRISPR-Cas9 with engineered immunosilenced human T cell epitopes
Source: Nat Commun. 2019 Apr 23;10:1842. doi: 10.1038/s41467-019-09693-x (PMC6478683; doi:10.1038/s41467-019-09693-x)
Supplement: Supplementary file 1 — Supplementary Information [file 41467_2019_9693_MOESM1_ESM.pdf]

Supplementary Information

Multifunctional CRISPR-Cas9 with engineered immunosilenced human T cell epitopes

Ferdosi et al.

Supplementary Table 1. Predicted Cas9 immunogenic T cell epitopes

				Binding			Protein Processing				
**Rank**	**Position**	**Sequence**	**Code**	**IEDB**	**NetMHC**	**Syfpeithi**	**IEDB**	**ANN**	S_b	S_i	$S_b.S_i$
1	988-997	YLNAVVGTAL	γ	1.25	21.5	24	0.27	0.02	0.068	0.975	0.002
2	1281-1290	ILADANLDKV		1.25	11.37	31	-0.06	-0.49	0.003	0.447	0.002
3	236-244	GLFGNLIAL	δ	0.6	10.12	29	1.15	1.04	0.020	0.900	0.002
4	240-248	NLIALSLGL	α	1.7	61.18	25	0.15	0.22	0.061	0.903	0.006
5	615-623	ILEDIVLTL	β	1.5	53.29	29	0.28	0.56	0.023	0.710	0.007
6	614-623	DILEDIVLTL		4.6	3105.79	28	-1.53	-1.02	0.063	0.888	0.007
7	719-727	SLHEHIANL		1.4	9.14	30	0.93	0.82	0.013	0.380	0.008
8	415-423	HLGELHAIL		4.4	276.73	25	-0.75	-0.81	0.071	0.876	0.009
9	300-308	ILLSDILRV		0.3	6.51	29	0.67	0.7	0.019	0.404	0.011
10	1086-1095	VLSMPQVNIV		3.65	178.87	26	-1.05	-1.43	0.059	0.758	0.014
11	719-728	SLHEHIANLA		4.7	60.17	19	-0.98	-1.74	0.126	0.890	0.014
12	1194-1203	LIIKLPKYSL		8.5	966.31	25	-0.97	-1.04	0.090	0.841	0.014
13	1346-1355	TLIHQSITGL		1.95	57.8	27	0.12	-0.06	0.043	0.632	0.016
14	1197-1207	KLPKYSLFEL		1.2	10.93	27	0.9	0.5	0.040	0.579	0.017
15	1041-1050	NIMNFFKTEI		2.65	314.8	19	-1.03	-0.9	0.121	0.857	0.017
16	512-520	SLLYEYFTV		0.4	4.56	25	0.67	0.55	0.056	0.678	0.018
17	1309-1318	IIHLFTLTNL		4.25	1083.6	24	-1.04	-0.78	0.085	0.787	0.018
18	661-670	RLSRKLINGI		3.5	278.03	24	-0.82	-1.05	0.078	0.746	0.020
19	1227-1236	ALPSKYVNFL		4.3	111.14	27	0.05	-0.26	0.051	0.594	0.021
20	996-1004	ALIKKYPKL		2.6	154.09	28	-0.27	0	0.037	0.407	0.022
21	221-229	RLENLIAQL		4.2	242.87	26	-0.46	-0.46	0.061	0.581	0.026
22	1237-1245	YLASHYEKL		1.2	10.3	26	0.9	0.84	0.050	0.446	0.027
23	1265-1273	YLDEIIEQI		0.3	4.8	26	0.62	0.6	0.046	0.399	0.028
24	1042-1050	IMNFFKTEI		3.2	131.4	21	-0.69	-0.87	0.103	0.724	0.028
25	815-824	YLQNGRDMYV		0.25	13.01	22	-0.18	-0.07	0.083	0.627	0.031
26	1212-1220	RMLASAGEL		3.2	333.2	22	-0.64	-0.51	0.095	0.650	0.033
27	1020-1029	KMIAKSEQEI		3.1	64.01	21	-0.36	-0.9	0.103	0.671	0.034
28	793-801	SQILKEHPV		2.8	191.23	16	-1.4	-1.36	0.149	0.766	0.035
29	742-750	KVVDELVKV		2.8	44.75	24	-0.06	-0.26	0.074	0.505	0.036
30	1181-1190	FLEAKGYKEV		3.25	105.27	21	-1.08	-1.42	0.103	0.651	0.036
31	160-169	HMIKFRGHFL		4.75	324.13	21	-0.59	-0.73	0.110	0.628	0.041
32	551-559	LLFKTNRKV		3	381.3	25	-1.52	-1.25	0.067	0.368	0.043
33	141-149	KLVDSTDKA		3.4	274.05	20	-1.48	-1.17	0.114	0.520	0.055
34	472-481	TITPWNFEEV		4.45	124.55	21	-0.84	-1.21	0.107	0.429	0.061
35	194-203	QLFEENPINA		1.65	67.94	17	-0.71	-0.79	0.135	0.469	0.072
36	518-527	FTVYNELTKV		2.55	169.93	20	-1.12	-1.15	0.111	0.216	0.087
37	473-481	ITPWNFEEV		6.4	351.14	18	-1.25	-1.65	0.143	0.229	0.110
38	970-978	FQFYKVREI		2.7	135.61	16	-0.66	-0.39	0.148	0.229	0.114

The table shows Cas9 HLA-A*02:01 epitopes predicted using an integrative prediction model and ranked according to their $S_b.S_i$ score (the lower the more immunogenic). The immunodominant and subdominant epitopes as confirmed by ELISpot are highlighted in dark gray and light gray, respectively. S_b, binding score; S_i, immunogenicity score.

Supplementary Table 2. Sequence homology of epitope α to amino acid sequences from known proteins

	Sequence	*Similarity (%)*	*Protein*	*Sequence ID*	*Source*
1	NLIALSLGL	9/9 (100%)	type II CRISPR RNA-guided endonuclease Cas9	WP_014612333.1	*Streptococcus dysgalactiae*
2	NLIALSLGL	9/9 (100%)	type II CRISPR RNA-guided endonuclease Cas9	WP_054279288.1	*Streptococcus phocae*
3	NLIALSLGL	9/9 (100%)	type II CRISPR RNA-guided endonuclease Cas9	WP_067062573.1	*Streptococcus pantholopis*
4	NLIALSLGL	9/9 (100%)	type II CRISPR RNA-guided endonuclease Cas9	WP_048800889.1	*Streptococcus constellatus*
5	NLIALSLGL	9/9 (100%)	type II CRISPR RNA-guided endonuclease Cas9	WP_002304487.1	*Streptococcus mutans*
6	NLIALSLGL	9/9 (100%)	type II CRISPR RNA-guided endonuclease Cas9	WP_049516684.1	*Streptococcus anginosus*
7	NLIALSLGL	9/9 (100%)	type II CRISPR RNA-guided endonuclease Cas9	WP_003079701.1	*Streptococcus macacae*
8	NLIALSLGL	9/9 (100%)	type II CRISPR RNA-guided endonuclease Cas9	GAD40915.1	*Streptococcus intermedius* SK54
9	NLIA**F**SLGL	8/9 (89%)	Full=RNA polymerase-associated protein RapA; AltName: Full=ATP-dependent helicase HepA	Q6LV34.1	Photobacterium profundum SS9
10	NLI**S**LSLGL	8/9 (89%)	type II CRISPR RNA-guided endonuclease Cas9	WP_096633625.1	*Streptococcus parauberis*
11	NLIAL**A**LGL	8/9 (89%)	type II CRISPR RNA-guided endonuclease Cas9	WP_075103982.1	*Streptococcus cuniculi*
12	NLIAL**A**LGL	8/9 (89%)	type II CRISPR RNA-guided endonuclease Cas9	WP_058692367.1	*Streptococcus gallolyticus*
13	NLIAL**A**LGL	8/9 (89%)	type II CRISPR RNA-guided endonuclease Cas9	WP_061100419.1	*Streptococcus pasteurianus*
14	NLIAL**A**LGL	8/9 (89%)	type II CRISPR RNA-guided endonuclease Cas9	WP_018363470.1	*Streptococcus caballi*
15	NLIAL**A**LGL	8/9 (89%)	type II CRISPR RNA-guided endonuclease Cas9	WP_099412266.1	*Streptococcus macedonicus*
16	NLIAL**A**LGL	8/9 (89%)	type II CRISPR RNA-guided endonuclease Cas9	WP_014334983.1	*Streptococcus infantarius*
17	**D**LIAL**Y**LGL	7/9 (78%)	Full=NADH-quinone oxidoreductase subunit N; AltName: Full=NADH dehydrogenase I subunit N; AltName: Full=NDH-1 subunit N	A8I421.1	Azorhizobium caulinodans ORS 571
18	NL**L**AL**A**LGL	7/9 (78%)	type II CRISPR RNA-guided endonuclease Cas9	WP_007896501.1	*Streptococcus pseudoporcinus*
19	NLI**G**L**A**LGL	7/9 (78%)	type II CRISPR RNA-guided endonuclease Cas9	WP_061587801.1	*Streptococcus oralis*
20	NL**V**AL**A**LGL	7/9 (78%)	type II CRISPR RNA-guided endonuclease Cas9	WP_074862269.1	*Streptococcus equinus*
21	NL**V**AL**V**LGL	7/9 (78%)	type II CRISPR RNA-guided endonuclease Cas9	WP_020917064.1	*Streptococcus lutetiensis*
22	**S**LIA**F**SLGL	7/9 (78%)	ectoine/hydroxyectoine ABC transporter permease subunit EhuD	WP_086160327.1	*Streptomyces* sp. SCSIO 03032
23	**Y**LIAL**A**LGL	7/9 (78%)	ectoine/hydroxyectoine ABC transporter permease subunit EhuD	WP_026413155.1	*Actinomadura oligospora*

Supplementary Table 3. Sequence homology of epitope β to amino acid sequences from known proteins

	Sequence	*Similarity (%)*	*Protein*	*Sequence ID*	*Source*
1	ILEDIVLTL	9/9 (100%)	type II CRISPR RNA-guided endonuclease Cas9	WP_084916602.1	*Streptococcus dysgalactiae*
2	ILEDIVLTL	9/9 (100%)	type II CRISPR RNA-guided endonuclease Cas9	WP_074484960.1	*Streptococcus henryi*
3	ILEDIVLTL	9/9 (100%)	type II CRISPR RNA-guided endonuclease Cas9	WP_003088697.1	*Streptococcus ratti*
4	ILEDIVLTL	9/9 (100%)	type II CRISPR RNA-guided endonuclease Cas9	WP_044681799.1	*Streptococcus suis*
5	ILEDIVLTL	9/9 (100%)	type II CRISPR RNA-guided endonuclease Cas9	WP_024786433.1	*Streptococcus mutans*
6	ILEDIVLTL	9/9 (100%)	type II CRISPR RNA-guided endonuclease Cas9	WP_057491067.1	*Streptococcus orisasini*
7	ILEDIVLTL	9/9 (100%)	type II CRISPR RNA-guided endonuclease Cas9	WP_082312238.1	*Streptococcus intermedius*
8	ILE**G**IVLTL	8/9 (89%)	peptide chain release factor 2	NP_275123.1	*Neisseria meningitidis* MC58
9	ILEDIV**Q**TL	8/9 (89%)	type II CRISPR RNA-guided endonuclease Cas9	EAO61901.1	*Streptococcus agalactiae*
10	ILEDIV**Q**TL	8/9 (89%)	type II CRISPR RNA-guided endonuclease Cas9	WP_070454905.1	*Streptococcus* sp. HMSC063D10
11	**V**LEDIVLTL	8/9 (89%)	type II CRISPR RNA-guided endonuclease Cas9	WP_075346866.1	*Streptococcus* sp. '*caviae*'
12	**V**LEDIVL**S**L	7/9 (78%)	type II CRISPR RNA-guided endonuclease Cas9	WP_093650272.1	*Streptococcus varani*
13	ILE**N**IV**H**TL	7/9 (78%)	type II CRISPR RNA-guided endonuclease Cas9	KYF37509.1	*Streptococcus mitis*
14	ILE**N**IV**H**TL	7/9 (78%)	type II CRISPR RNA-guided endonuclease Cas9	WP_084972088.1	*Streptococcus oralis*
15	ILE**N**IV**H**TL	7/9 (78%)	type II CRISPR RNA-guided endonuclease Cas9	WP_045635197.1	*Streptococcus gordonii*

Supplementary Table 4. Multiple comparison results of mutated epitope ELISpot screening

Donor ID	p value	Corrected p value
Peptide α		
5	0.0000003	0.000001
10	0.0000075	0.0000124
9	0.0007779	0.0008557
1	0.0017641	0.0014554
12	0.0079662	0.0052577
7	0.0387297	0.0213013
6	0.045632	0.0215122
2	0.0655837	0.0270533
3	0.0783807	0.0287396
Peptide β		
9	0.0002026	0.0005757
6	0.0003655	0.0005757
7	0.0007587	0.0007967
3	0.005237	0.0041241
2	0.0096527	0.0060812
1	0.0219659	0.0099651
10	0.0221447	0.0099651
12	0.0272351	0.0107238

Uncorrected and corrected p values for donors with a significant reduction in T cell response after mutating epitope α or β by the Benjamini-Hochberg method (5% FDR) are shown for Supplementary Figure 1.

Supplementary Table 5. Sequences of SpCas9 non-HLA-A*02:01 class I epitopes used to screen for pre-existing T cell reactivity against SpCas9 in healthy donors

Peptide ID	Peptide Sequence	HLA Allele(s)
**15**	KTYAHLFDDK	A*03:01& 11:01
**16**	FLYLASHYEK	A*03:01 & 24:02 & B55:01 & B08:01
**17**	KTNRKVTVK	A*03:01
**18**	KVLPKHSLLY	A*03:01
**19**	AILSARLSK	A*03:01 & 11:01
**20**	LSMPQVNIVK	A*11:01 & 24:02 & B44:02 & B44:03
**21**	TIMERSSFEK	A*11:01 & B08:01 & B55:01
**22**	YSNIMNFFK	A*11:01 & 24:02
**23**	KYPKLESEF	A*24:02 & B08:01
**24**	RYTRRKNRI	A*24:02
**25**	YFFYSNIMNF	A*24:02
**26**	ELDINRLSDY	A*01:01
**27**	NLDKVLSAY	A*01:01
**28**	ESEFVYGDY	A*01:01
**29**	ILDSRMNTKY	A*01:01 & B08:01
**30**	NTQLQNEKLY	A*01:01
**31**	NELALPSKY	B*44:02 & B44:03
**32**	NEMAKVDDSF	B*44:02 & B55:01 & B08:01 & B44:03
**33**	SEETITPWNF	B*44:02 & B44:03
**34**	EETITPWNF	B*44:02 & B08:01 & B44:03
**35**	IANLAGSPA	B*55:01 & B44:03

Supplementary Table 6. Sequences of long peptides that include epitopes in the top 2% of predicted MHC class II binders that were used to screen for SpCas9 class II immune reactivity

Peptide ID	Peptide Sequence	HLA
**1**	ILEDIVLTLTLFEDR	DPA1/DPB2
**2**	KNGLFGNLIALSLGLTPNF	DRB1
**3**	RLSDYDVAAIVPQSFLK	DQA1
**4**	ERHPIFGNIVDEVAYHY	DQA1-2
**5**	VLPKHSLLYEYFTVYNELT	DPA1
**6**	ATAKYFFYSNIMNFFK	DPA1-2
**7**	DLRLIYLALAHMIKFR	DRB1-2
**8**	MKNYWRQLLNAKLITQ	DRB1-3

Sequences of peptides α and β are highlighted.

Supplementary Table 7. Sequences of primers used in this study

**Cas9 fragment1- FW**	ttttGGTCTCTAGGTCCACCATGGACTATAAGGACCACGA
**Cas9 fragment1- RV**	tttggtctcaGAACAGCTGGTTGTAGGTCTGCA
**Cas9 fragment2-FW**	ttttGGTCTCTACCAACCGGAAAGTGACCGTGAAG
**Cas9 fragment2-RV**	ttttGGTCTCAAAGCTTACTTTTTCTTTTTTGCC
**qPCRMIAT-FW**	TGGCTGGGGTTTGAACCTTT
**qPCR-MIAT RV**	AGGAAGCTGTTCCAGACTGC
**qPCRTTN FW**	TGTTGCCACTGGTGCTAAAG
**qPCR-TTN-RV**	ACAGCAGTCTTCTCCGCTTC
**PCR-EMX1-FW**	CCATCCCCTTCTGTGAATGT
**PCR-EMX1-RV**	GGAGATTGGAGACACGGAGA

Supplementary Table 8. Sequences of gRNAs used in this study

**MIAT-14bp gRNA**	GAGGCTGAGCGCAC
**TTN-14bp gRNA**	GGAAGTCTCCTTTG
**Reporter2-20bp gRNA**	GTCCCCTCCACCCCACAGTG
**CR10-14bp-gRNA**	GCATCAGGAACATGT
**EMX1- 20bp gRNA**	CACC GAGTCCGAGCAGAAGAAGAA

Supplementary Figure 1

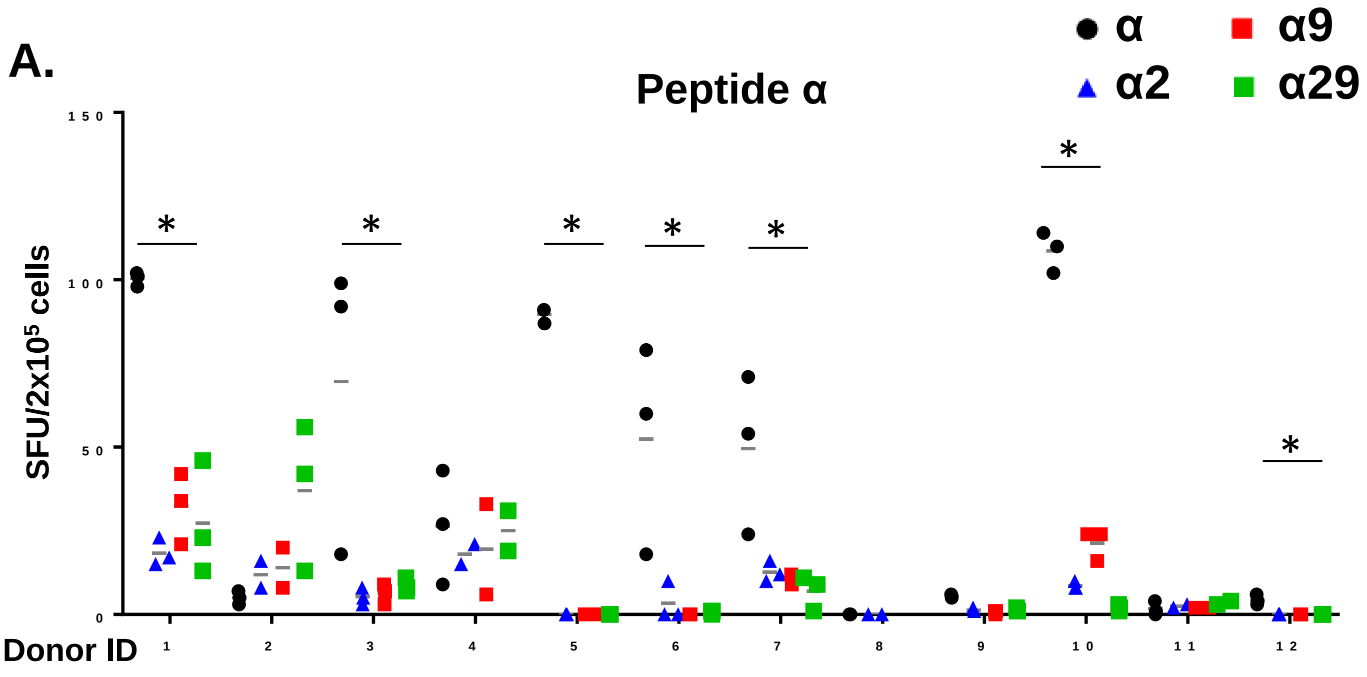

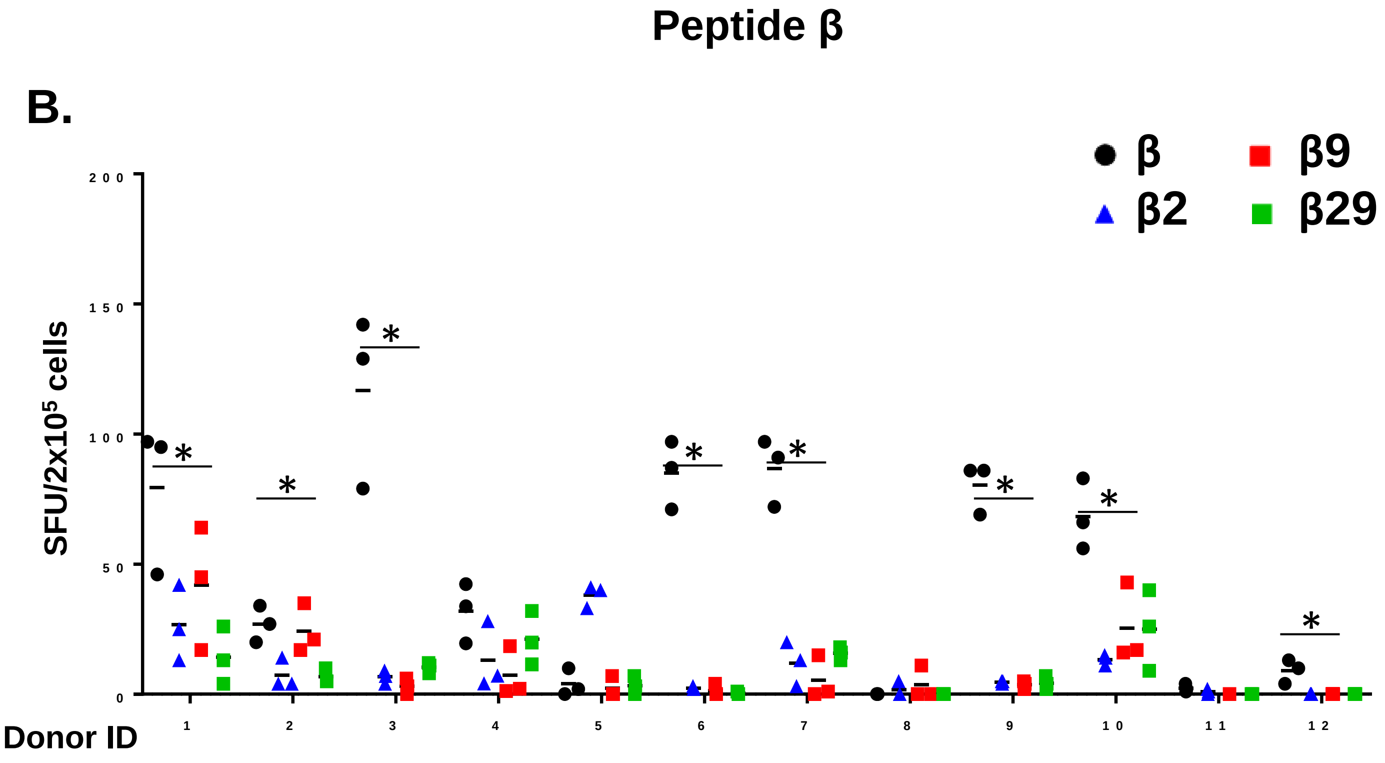

Supplementary Figure 1. Reduced T cell response to epitopes α and β after mutation of the anchor residues. **A and B.** IFN-γ ELISpot for 12 healthy donor PBMCs stimulated with wild type or mutated peptide α (**A**) or β (**B**). *Significant SFU change (corrected p value<0.03, 5% FDR; see Supplementary Table 4). Statistical analysis was performed post hoc and results are exploratory.

Supplementary Figure 2

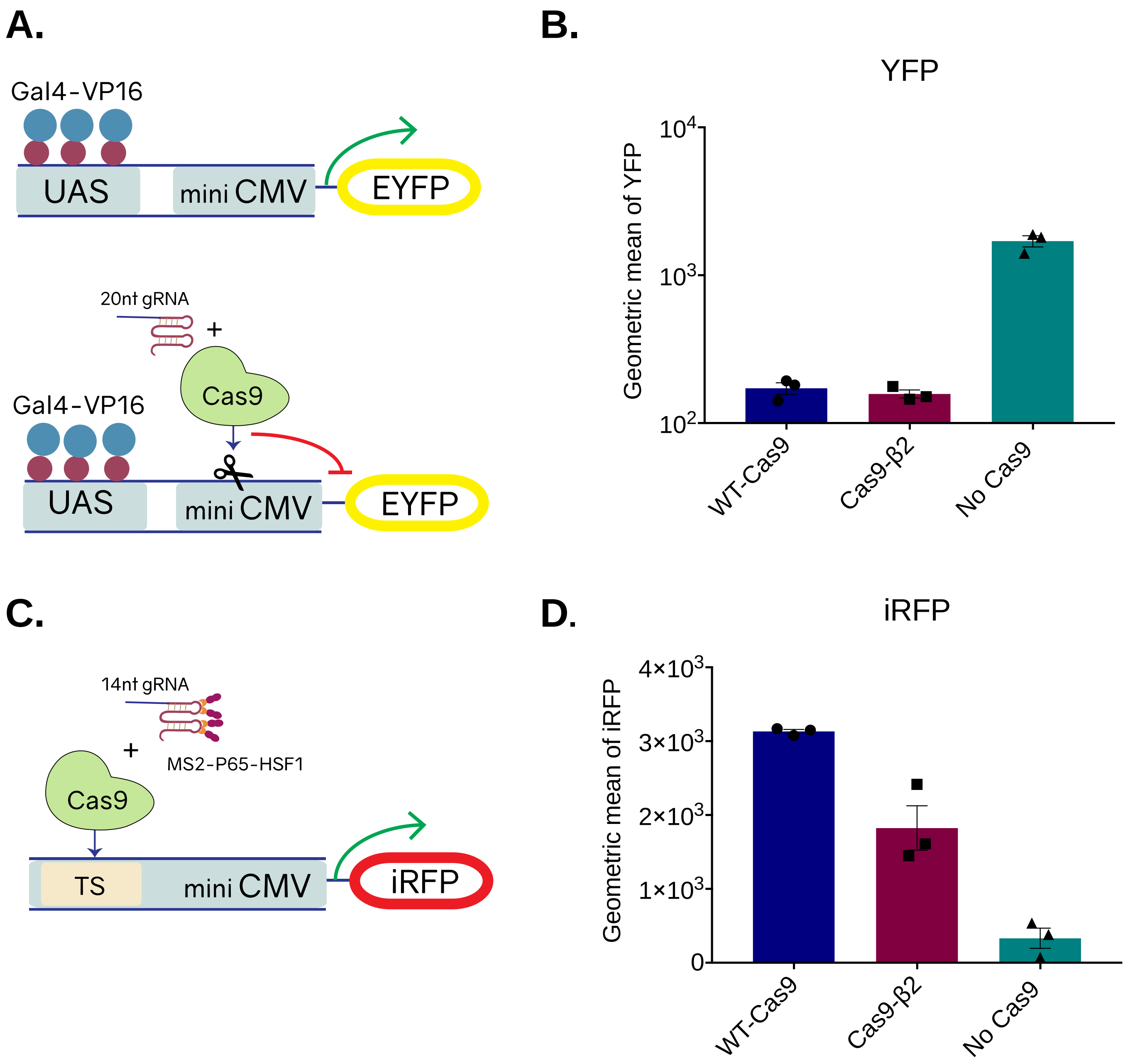

Supplementary Figure 2. A. Schematic of the experiment assessing Cas9-β2 cleavage capacity at a synthetic promoter. Cells were transfected with either WT-Cas9, Cas9-β2 or an empty plasmid as well as 20nt gRNA targeting a synthetic CRISPR promoter that harbors two gRNA target sites flanking a mini-CMV promoter. The targeting and cleavage at the promoter should disrupt the promoter and decrease EYFP expression. **B.** Each individual dot represents EYFP expression 48 hours after transfection in cells expressing >2x10^2 A.U. of a transfection marker (BFP) measured by flow cytometry (n=3 independent technical replicates). **C.** Schematic of the experiment assessing Cas9-β2 transcriptional activation capacity at a synthetic promoter. Cells were transfected with either WT-Cas9, Cas9-β2 or an empty plasmid as well as aptamer binding transcriptional activation domains, and a 14nt gRNA targeting a synthetic CRISPR promoter that harbors multiple target sites upstream of a mini-CMV promoter. Targeting the promoter should enable iRFP expression. **D.** Each individual dot shows iRFP expression 48 hours after transfection in cells expressing >2x10^2 A.U. of a transfection marker (GFP) measured by flow cytometry (n=3 independent technical replicates). Data are presented as mean ± S.E.M.

Supplementary Figure 3

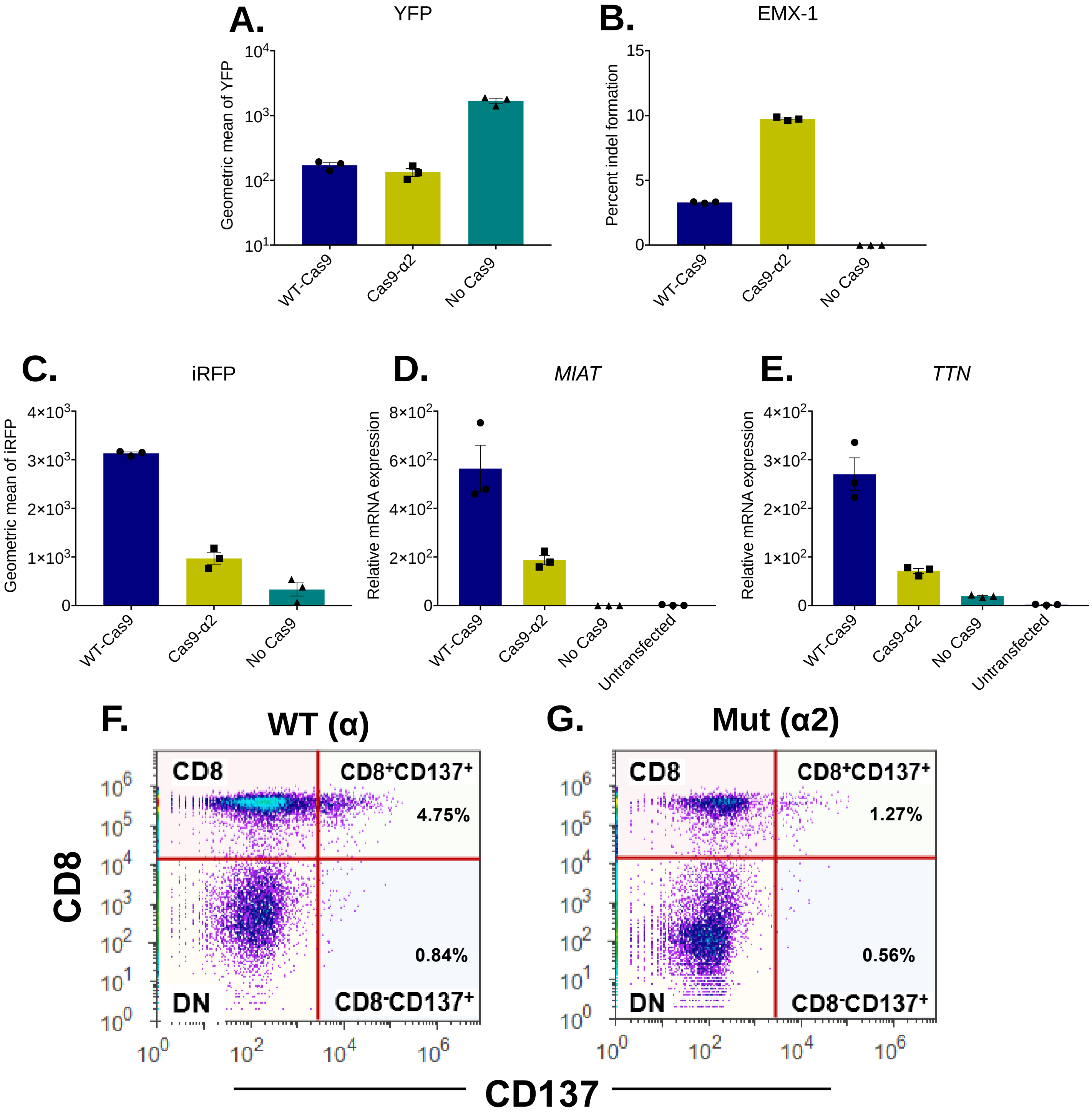

Supplementary Figure 3. A. Analysis of cleavage capacity of Cas9-α2 as compared to WT-Cas9 in a synthetic promoter. Each individual dot shows EYFP expression 48 hours after transfection in cells expressing >2x10^2 A.U. of a transfection marker measured by flow cytometry (n=3 independent technical replicates). **B.** Percentage of indel formation in *EMX-1* locus (n=3 independent technical replicates). **C.** Transcriptional modulation by Cas9-α2 at a synthetic promoter. Each individual dot shows iRFP expression 48 hours after transfection in cells expressing >2x10^2 A.U. of a transfection marker measured by flow cytometry (n=3 independent technical replicates). **D, E.** Data represent the mRNA level relative to an untransfected control experiment. Note for A and C: WT-Cas9 and no Cas9 data are also reported in Supplementary Figure 2. For B-E: WT-Cas9 and no Cas9 data are also reported in Fig.3. (n=3 independent technical replicates). Data represent mean ± S.E.M. **F.** Activated CD8+CD137+ T cells detected in PBMCs stimulated with peptide α were reduced in PBMCs stimulated with peptide α2 (data representative of 3 individual stimulations).

Supplementary Figure 4

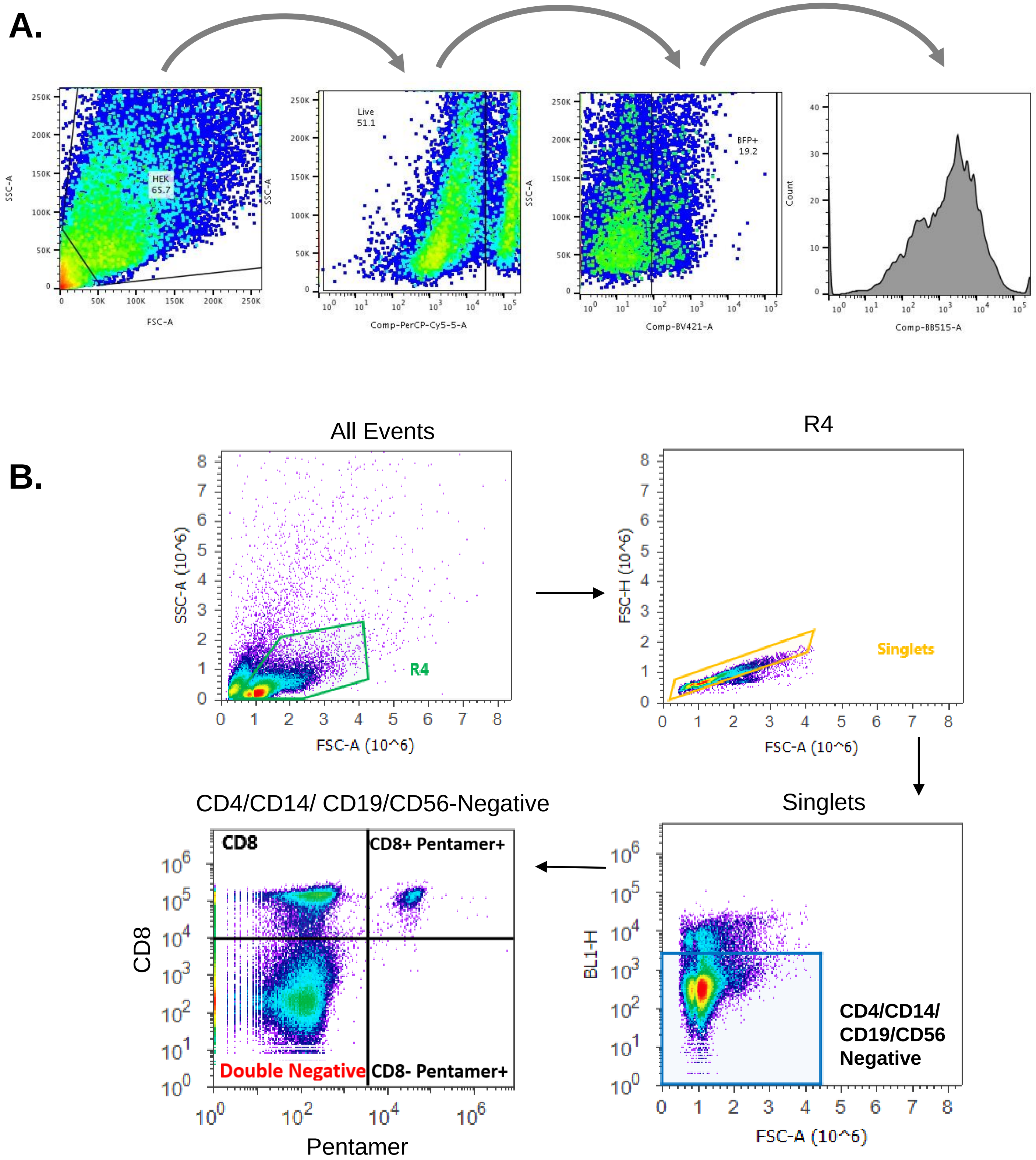

Supplementary Figure 4. A. Representative flow cytometry gating for analysis of Cas9 function on synthetic promoters (Supplementary Figures 2 and 3A,C). Cells are gated based on Forward (FSC) and Side Scatter (SSCs). Live cells (PerCP negative population) are selected and then the transfected population is gated based on the expression of a transfection control (here BFP-BV421) more than $2x10^2$ A.U. The geometric mean of the output (here YFP-BB515) is determined in this population. **B.** Flow cytometry gating for analysis of Cas9 pentamer+ CD8+ T lymphocytes (Fig. 2A, B). Cells are gated based on FSC and SSC and negatively gated on CD4/CD14/CD19/CD56. The CD8-, CD8+, and Cas9 pentamer+ population are shown (bottom right).

Supplementary Note 1

Sequences of modified Cas9 genes.

Cas9-α2

ATGGACTATAAGGACCACGACGGAGACTACAAGGATCATGATATTGATTACAAAGACGA
TGACGATAAGATGGCCCCAAAGAAGAAGCGGAAGGTCGGTATCCACGGAGTCCCAGC
AGCCGACAAGAAGTACAGCATCGGCCTGGACATCGGCACCAACTCTGTGGGCTGGGC
CGTGATCACCGACGAGTACAAGGTGCCCAGCAAGAAATTCAAGGTGCTGGGCAACACC
GACCGGCACAGCATCAAGAAGAACCTGATCGGAGCCCTGCTGTTCGACAGCGGCGAA
ACAGCCGAGGCCACCCGGCTGAAGAGAACCGCCAGAAGAAGATACACCAGACGGAAG
AACCGGATCTGCTATCTGCAAGAGATCTTCAGCAACGAGATGGCCAAGGTGGACGACA
GCTTCTTCCACAGACTGGAAGAGTCCTTCCTGGTGGAAGAGGATAAGAAGCACGAGCG
GCACCCCATCTTCGGCAACATCGTGGACGAGGTGGCCTACCACGAGAAGTACCCCAC
CATCTACCACCTGAGAAAGAAACTGGTGGACAGCACCGACAAGGCCGACCTGCGGCT
GATCTATCTGGCCCTGGCCCACATGATCAAGTTCCGGGGCCACTTCCTGATCGAGGGC
GACCTGAACCCCGACAACAGCGACGTGGACAAGCTGTTCATCCAGCTGGTGCAGACCT
ACAACCAGCTGTTCGAGGAAAACCCCATCAACGCCAGCGGCGTGGACGCCAAGGCCA
TCCTGTCTGCCAGACTGAGCAAGAGCAGACGGCTGGAAAATCTGATCGCCCAGCTGCC
CGGCGAGAAGAAGAATGGCCTGTTCGGAAACGGTATTGCCCTGAGCCTGGGCCTGAC
CCCCAACTTCAAGAGCAACTTCGACCTGGCCGAGGATGCCAAACTGCAGCTGAGCAAG
GACACCTACGACGACGACCTGGACAACCTGCTGGCCCAGATCGGCGACCAGTACGCC
GACCTGTTTCTGGCCGCCAAGAACCTGTCCGACGCCATCCTGCTGAGCGACATCCTGA
GAGTGAACACCGAGATCACCAAGGCCCCCCTGAGCGCCTCTATGATCAAGAGATACGA
CGAGCACCACCAGGACCTGACCCTGCTGAAAGCTCTCGTGCGGCAGCAGCTGCCTGA
GAAGTACAAAGAGATTTTCTTCGACCAGAGCAAGAACGGCTACGCCGGCTACATTGAC
GGCGGAGCCAGCCAGGAAGAGTTCTACAAGTTCATCAAGCCCATCCTGGAAAAGATGG
ACGGCACCGAGGAACTGCTCGTGAAGCTGAACAGAGAGGACCTGCTGCGGAAGCAGC
GGACCTTCGACAACGGCAGCATCCCCCACCAGATCCACCTGGGAGAGCTGCACGCCA
TTCTGCGGCGGCAGGAAGATTTTTACCCATTCCTGAAGGACAACCGGGAAAAGATCGA
GAAGATCCTGACCTTCCGCATCCCCTACTACGTGGGCCCTCTGGCCAGGGGAAACAG
CAGATTCGCCTGGATGACCAGAAAGAGCGAGGAAACCATCACCCCCTGGAACTTCGAG
GAAGTGGTGGACAAGGGCGCTTCCGCCCAGAGCTTCATCGAGCGGATGACCAACTTC
GATAAGAACCTGCCCAACGAGAAGGTGCTGCCCAAGCACAGCCTGCTGTACGAGTACT
TCACCGTGTATAACGAGCTGACCAAAGTGAAATACGTGACCGAGGGAATGAGAAAGCC
CGCCTTCCTGAGCGGCGAGCAGAAAAAGGCCATCGTGGACCTGCTGTTCAAGACCAA
CCGGAAAGTGACCGTGAAGCAGCTGAAAGAGGACTACTTCAAGAAAATCGAGTGCTTC
GACTCCGTGGAAATCTCCGGCGTGGAAGATCGGTTCAACGCCTCCCTGGGCACATACC
ACGATCTGCTGAAAATTATCAAGGACAAGGACTTCCTGGACAATGAGGAAAACGAGGA
CATTCTTGAAGATATCGTGCTGACCCTGACACTGTTTGAGGACAGAGAGATGATCGAG
GAACGGCTGAAAACCTATGCCCACCTGTTCGACGACAAAGTGATGAAGCAGCTGAAGC
GGCGGAGATACACCGGCTGGGGCAGGCTGAGCCGGAAGCTGATCAACGGCATCCGG
GACAAGCAGTCCGGCAAGACAATCCTGGATTTCCTGAAGTCCGACGGCTTCGCCAACA
GAAACTTCATGCAGCTGATCCACGACGACAGCCTGACCTTTAAAGAGGACATCCAGAA
AGCCCAGGTGTCCGGCCAGGGCGATAGCCTGCACGAGCACATTGCCAATCTGGCCGG
CAGCCCCGCCATTAAGAAGGGCATCCTGCAGACAGTGAAGGTGGTGGACGAGCTCGT
GAAAGTGATGGGCCGGCACAAGCCCGAGAACATCGTGATCGAAATGGCCAGAGAGAA
CCAGACCACCCAGAAGGGACAGAAGAACAGCCGCGAGAGAATGAAGCGGATCGAAGA
GGGCATCAAAGAGCTGGGCAGCCAGATCCTGAAAGAACACCCCGTGGAAAACACCCA

GCTGCAGAACGAGAAGCTGTACCTGTACTACCTGCAGAATGGGCGGGATATGTACGTG
GACCAGGAACTGGACATCAACCGGCTGTCCGACTACGATGTGGACCATATCGTGCCTC
AGAGCTTTCTGAAGGACGACTCCATCGACAACAAGGTGCTGACCAGAAGCGACAAGAA
CCGGGGCAAGAGCGACAACGTGCCCTCCGAAGAGGTCGTGAAGAAGATGAAGAACTA
CTGGCGGCAGCTGCTGAACGCCAAGCTGATTACCCAGAGAAAGTTCGACAATCTGACC
AAGGCCGAGAGAGGCGGCCTGAGCGAACTGGATAAGGCCGGCTTCATCAAGAGACAG
CTGGTGGAAACCCGGCAGATCACAAAGCACGTGGCACAGATCCTGGACTCCCGGATG
AACACTAAGTACGACGAGAATGACAAGCTGATCCGGGAAGTGAAAGTGATCACCCTGA
AGTCCAAGCTGGTGTCCGATTTCCGGAAGGATTTCCAGTTTTACAAAGTGCGCGAGAT
CAACAACTACCACCACGCCCACGACGCCTACCTGAACGCCGTCGTGGGAACCGCCCT
GATCAAAAAGTACCCTAAGCTGGAAAGCGAGTTCGTGTACGGCGACTACAAGGTGTAC
GACGTGCGGAAGATGATCGCCAAGAGCGAGCAGGAAATCGGCAAGGCTACCGCCAAG
TACTTCTTCTACAGCAACATCATGAACTTTTTCAAGACCGAGATTACCCTGGCCAACGG
CGAGATCCGGAAGCGGCCTCTGATCGAGACAAACGGCGAAACCGGGGAGATCGTGTG
GGATAAGGGCCGGGATTTTGCCACCGTGCGGAAAGTGCTGAGCATGCCCCAAGTGAA
TATCGTGAAAAAGACCGAGGTGCAGACAGGCGGCTTCAGCAAAGAGTCTATCCTGCCC
AAGAGGAACAGCGATAAGCTGATCGCCAGAAAGAAGGACTGGGACCCTAAGAAGTAC
GGCGGCTTCGACAGCCCCACCGTGGCCTATTCTGTGCTGGTGGTGGCCAAAGTGGAA
AAGGGCAAGTCCAAGAAACTGAAGAGTGTGAAAGAGCTGCTGGGGATCACCATCATGG
AAAGAAGCAGCTTCGAGAAGAATCCCATCGACTTTCTGGAAGCCAAGGGCTACAAAGA
AGTGAAAAAGGACCTGATCATCAAGCTGCCTAAGTACTCCCTGTTCGAGCTGGAAAAC
GGCCGGAAGAGAATGCTGGCCTCTGCCGGCGAACTGCAGAAGGGAAACGAACTGGCC
CTGCCCTCCAAATATGTGAACTTCCTGTACCTGGCCAGCCACTATGAGAAGCTGAAGG
GCTCCCCCGAGGATAATGAGCAGAAACAGCTGTTTGTGGAACAGCACAAGCACTACCT
GGACGAGATCATCGAGCAGATCAGCGAGTTCTCCAAGAGAGTGATCCTGGCCGACGC
TAATCTGGACAAAGTGCTGTCCGCCTACAACAAGCACCGGGATAAGCCCATCAGAGAG
CAGGCCGAGAATATCATCCACCTGTTTACCCTGACCAATCTGGGAGCCCCTGCCGCCT
TCAAGTACTTTGACACCACCATCGACCGGAAGAGGTACACCAGCACCAAAGAGGTGCT
GGACGCCACCCTGATCCACCAGAGCATCACCGGCCTGTACGAGACACGGATCGACCT
GTCTCAGCTGGGAGGCGACAAAAGGCCGGCGGCCACGAAAAAGGCCGGCCAGGCAA
AAAAGAAAAAGTAAG

Cas9-β2

ATGGACTATAAGGACCACGACGGAGACTACAAGGATCATGATATTGATTACAAAGACGA
TGACGATAAGATGGCCCCAAAGAAGAAGCGGAAGGTCGGTATCCACGGAGTCCCAGC
AGCCGACAAGAAGTACAGCATCGGCCTGGACATCGGCACCAACTCTGTGGGCTGGGC
CGTGATCACCGACGAGTACAAGGTGCCCAGCAAGAAATTCAAGGTGCTGGGCAACACC
GACCGGCACAGCATCAAGAAGAACCTGATCGGAGCCCTGCTGTTCGACAGCGGCGAA
ACAGCCGAGGCCACCCGGCTGAAGAGAACCGCCAGAAGAAGATACACCAGACGGAAG
AACCGGATCTGCTATCTGCAAGAGATCTTCAGCAACGAGATGGCCAAGGTGGACGACA
GCTTCTTCCACAGACTGGAAGAGTCCTTCCTGGTGGAAGAGGATAAGAAGCACGAGCG
GCACCCCATCTTCGGCAACATCGTGGACGAGGTGGCCTACCACGAGAAGTACCCCAC
CATCTACCACCTGAGAAAGAAACTGGTGGACAGCACCGACAAGGCCGACCTGCGGCT
GATCTATCTGGCCCTGGCCCACATGATCAAGTTCCGGGGCCACTTCCTGATCGAGGGC
GACCTGAACCCCGACAACAGCGACGTGGACAAGCTGTTCATCCAGCTGGTGCAGACCT
ACAACCAGCTGTTCGAGGAAAACCCCATCAACGCCAGCGGCGTGGACGCCAAGGCCA
TCCTGTCTGCCAGACTGAGCAAGAGCAGACGGCTGGAAAATCTGATCGCCCAGCTGCC
CGGCGAGAAGAAGAATGGCCTGTTCGGAAACCTTATTGCCCTGAGCCTGGGCCTGAC
CCCCAACTTCAAGAGCAACTTCGACCTGGCCGAGGATGCCAAACTGCAGCTGAGCAAG
GACACCTACGACGACGACCTGGACAACCTGCTGGCCCAGATCGGCGACCAGTACGCC

GACCTGTTTCTGGCCGCCAAGAACCTGTCCGACGCCATCCTGCTGAGCGACATCCTGA
GAGTGAACACCGAGATCACCAAGGCCCCCCTGAGCGCCTCTATGATCAAGAGATACGA
CGAGCACCACCAGGACCTGACCCTGCTGAAAGCTCTCGTGCGGCAGCAGCTGCCTGA
GAAGTACAAAGAGATTTTCTTCGACCAGAGCAAGAACGGCTACGCCGGCTACATTGAC
GGCGGAGCCAGCCAGGAAGAGTTCTACAAGTTCATCAAGCCCATCCTGGAAAAGATGG
ACGGCACCGAGGAACTGCTCGTGAAGCTGAACAGAGAGGACCTGCTGCGGAAGCAGC
GGACCTTCGACAACGGCAGCATCCCCCACCAGATCCACCTGGGAGAGCTGCACGCCA
TTCTGCGGCGGCAGGAAGATTTTTACCCATTCCTGAAGGACAACCGGGAAAAGATCGA
GAAGATCCTGACCTTCCGCATCCCCTACTACGTGGGCCCTCTGGCCAGGGGAAACAG
CAGATTCGCCTGGATGACCAGAAAGAGCGAGGAAACCATCACCCCCTGGAACTTCGAG
GAAGTGGTGGACAAGGGCGCTTCCGCCCAGAGCTTCATCGAGCGGATGACCAACTTC
GATAAGAACCTGCCCAACGAGAAGGTGCTGCCCAAGCACAGCCTGCTGTACGAGTACT
TCACCGTGTATAACGAGCTGACCAAAGTGAAATACGTGACCGAGGGAATGAGAAAGCC
CGCCTTCCTGAGCGGCGAGCAGAAAAAGGCCATCGTGGACCTGCTGTTCAAGACCAA
CCGGAAAGTGACCGTGAAGCAGCTGAAAGAGGACTACTTCAAGAAAATCGAGTGCTTC
GACTCCGTGGAAATCTCCGGCGTGGAAGATCGGTTCAACGCCTCCCTGGGCACATACC
ACGATCTGCTGAAAATTATCAAGGACAAGGACTTCCTGGACAATGAGGAAAACGAGGA
CATTGGTGAAGATATCGTGCTGACCCTGACACTGTTTGAGGACAGAGAGATGATCGAG
GAACGGCTGAAAACCTATGCCCACCTGTTCGACGACAAAGTGATGAAGCAGCTGAAGC
GGCGGAGATACACCGGCTGGGGCAGGCTGAGCCGGAAGCTGATCAACGGCATCCGG
GACAAGCAGTCCGGCAAGACAATCCTGGATTTCCTGAAGTCCGACGGCTTCGCCAACA
GAAACTTCATGCAGCTGATCCACGACGACAGCCTGACCTTTAAAGAGGACATCCAGAA
AGCCCAGGTGTCCGGCCAGGGCGATAGCCTGCACGAGCACATTGCCAATCTGGCCGG
CAGCCCCGCCATTAAGAAGGGCATCCTGCAGACAGTGAAGGTGGTGGACGAGCTCGT
GAAAGTGATGGGCCGGCACAAGCCCGAGAACATCGTGATCGAAATGGCCAGAGAGAA
CCAGACCACCCAGAAGGGACAGAAGAACAGCCGCGAGAGAATGAAGCGGATCGAAGA
GGGCATCAAAGAGCTGGGCAGCCAGATCCTGAAAGAACACCCCGTGGAAAACACCCA
GCTGCAGAACGAGAAGCTGTACCTGTACTACCTGCAGAATGGGCGGGATATGTACGTG
GACCAGGAACTGGACATCAACCGGCTGTCCGACTACGATGTGGACCATATCGTGCCTC
AGAGCTTTCTGAAGGACGACTCCATCGACAACAAGGTGCTGACCAGAAGCGACAAGAA
CCGGGGCAAGAGCGACAACGTGCCCTCCGAAGAGGTCGTGAAGAAGATGAAGAACTA
CTGGCGGCAGCTGCTGAACGCCAAGCTGATTACCCAGAGAAAGTTCGACAATCTGACC
AAGGCCGAGAGAGGCGGCCTGAGCGAACTGGATAAGGCCGGCTTCATCAAGAGACAG
CTGGTGGAAACCCGGCAGATCACAAAGCACGTGGCACAGATCCTGGACTCCCGGATG
AACACTAAGTACGACGAGAATGACAAGCTGATCCGGGAAGTGAAAGTGATCACCCTGA
AGTCCAAGCTGGTGTCCGATTTCCGGAAGGATTTCCAGTTTTACAAAGTGCGCGAGAT
CAACAACTACCACCACGCCCACGACGCCTACCTGAACGCCGTCGTGGGAACCGCCCT
GATCAAAAAGTACCCTAAGCTGGAAAGCGAGTTCGTGTACGGCGACTACAAGGTGTAC
GACGTGCGGAAGATGATCGCCAAGAGCGAGCAGGAAATCGGCAAGGCTACCGCCAAG
TACTTCTTCTACAGCAACATCATGAACTTTTTCAAGACCGAGATTACCCTGGCCAACGG
CGAGATCCGGAAGCGGCCTCTGATCGAGACAAACGGCGAAACCGGGGAGATCGTGTG
GGATAAGGGCCGGGATTTTGCCACCGTGCGGAAAGTGCTGAGCATGCCCCAAGTGAA
TATCGTGAAAAAGACCGAGGTGCAGACAGGCGGCTTCAGCAAAGAGTCTATCCTGCCC
AAGAGGAACAGCGATAAGCTGATCGCCAGAAAGAAGGACTGGGACCCTAAGAAGTAC
GGCGGCTTCGACAGCCCCACCGTGGCCTATTCTGTGCTGGTGGTGGCCAAAGTGGAA
AAGGGCAAGTCCAAGAAACTGAAGAGTGTGAAAGAGCTGCTGGGGATCACCATCATGG
AAAGAAGCAGCTTCGAGAAGAATCCCATCGACTTTCTGGAAGCCAAGGGCTACAAAGA
AGTGAAAAAGGACCTGATCATCAAGCTGCCTAAGTACTCCCTGTTCGAGCTGGAAAAC
GGCCGGAAGAGAATGCTGGCCTCTGCCGGCGAACTGCAGAAGGGAAACGAACTGGCC
CTGCCCTCCAAATATGTGAACTTCCTGTACCTGGCCAGCCACTATGAGAAGCTGAAGG
GCTCCCCCGAGGATAATGAGCAGAAACAGCTGTTTGTGGAACAGCACAAGCACTACCT

GGACGAGATCATCGAGCAGATCAGCGAGTTCTCCAAGAGAGTGATCCTGGCCGACGC
TAATCTGGACAAAGTGCTGTCCGCCTACAACAAGCACCGGGATAAGCCCATCAGAGAG
CAGGCCGAGAATATCATCCACCTGTTTACCCTGACCAATCTGGGAGCCCCTGCCGCCT
TCAAGTACTTTGACACCACCATCGACCGGAAGAGGTACACCAGCACCAAAGAGGTGCT
GGACGCCACCCTGATCCACCAGAGCATCACCGGCCTGTACGAGACACGGATCGACCT
GTCTCAGCTGGGAGGCGACAAAAGGCCGGCGGCCACGAAAAAGGCCGGCCAGGCAA
AAAAGAAAAAGTAA

Cas9-α2-β2

ATGGACTATAAGGACCACGACGGAGACTACAAGGATCATGATATTGATTACAAAGACGA
TGACGATAAGATGGCCCCAAAGAAGAAGCGGAAGGTCGGTATCCACGGAGTCCCAGC
AGCCGACAAGAAGTACAGCATCGGCCTGGACATCGGCACCAACTCTGTGGGCTGGGC
CGTGATCACCGACGAGTACAAGGTGCCCAGCAAGAAATTCAAGGTGCTGGGCAACACC
GACCGGCACAGCATCAAGAAGAACCTGATCGGAGCCCTGCTGTTCGACAGCGGCGAA
ACAGCCGAGGCCACCCGGCTGAAGAGAACCGCCAGAAGAAGATACACCAGACGGAAG
AACCGGATCTGCTATCTGCAAGAGATCTTCAGCAACGAGATGGCCAAGGTGGACGACA
GCTTCTTCCACAGACTGGAAGAGTCCTTCCTGGTGGAAGAGGATAAGAAGCACGAGCG
GCACCCCATCTTCGGCAACATCGTGGACGAGGTGGCCTACCACGAGAAGTACCCCAC
CATCTACCACCTGAGAAAGAAACTGGTGGACAGCACCGACAAGGCCGACCTGCGGCT
GATCTATCTGGCCCTGGCCCACATGATCAAGTTCCGGGGCCACTTCCTGATCGAGGGC
GACCTGAACCCCGACAACAGCGACGTGGACAAGCTGTTCATCCAGCTGGTGCAGACCT
ACAACCAGCTGTTCGAGGAAAACCCCATCAACGCCAGCGGCGTGGACGCCAAGGCCA
TCCTGTCTGCCAGACTGAGCAAGAGCAGACGGCTGGAAAATCTGATCGCCCAGCTGCC
CGGCGAGAAGAAGAATGGCCTGTTCGGAAACGGTATTGCCCTGAGCCTGGGCCTGAC
CCCCAACTTCAAGAGCAACTTCGACCTGGCCGAGGATGCCAAACTGCAGCTGAGCAAG
GACACCTACGACGACGACCTGGACAACCTGCTGGCCCAGATCGGCGACCAGTACGCC
GACCTGTTTCTGGCCGCCAAGAACCTGTCCGACGCCATCCTGCTGAGCGACATCCTGA
GAGTGAACACCGAGATCACCAAGGCCCCCCTGAGCGCCTCTATGATCAAGAGATACGA
CGAGCACCACCAGGACCTGACCCTGCTGAAAGCTCTCGTGCGGCAGCAGCTGCCTGA
GAAGTACAAAGAGATTTTCTTCGACCAGAGCAAGAACGGCTACGCCGGCTACATTGAC
GGCGGAGCCAGCCAGGAAGAGTTCTACAAGTTCATCAAGCCCATCCTGGAAAAGATGG
ACGGCACCGAGGAACTGCTCGTGAAGCTGAACAGAGAGGACCTGCTGCGGAAGCAGC
GGACCTTCGACAACGGCAGCATCCCCCACCAGATCCACCTGGGAGAGCTGCACGCCA
TTCTGCGGCGGCAGGAAGATTTTTACCCATTCCTGAAGGACAACCGGGAAAAGATCGA
GAAGATCCTGACCTTCCGCATCCCCTACTACGTGGGCCCTCTGGCCAGGGGAAACAG
CAGATTCGCCTGGATGACCAGAAAGAGCGAGGAAACCATCACCCCCTGGAACTTCGAG
GAAGTGGTGGACAAGGGCGCTTCCGCCCAGAGCTTCATCGAGCGGATGACCAACTTC
GATAAGAACCTGCCCAACGAGAAGGTGCTGCCCAAGCACAGCCTGCTGTACGAGTACT
TCACCGTGTATAACGAGCTGACCAAAGTGAAATACGTGACCGAGGGAATGAGAAAGCC
CGCCTTCCTGAGCGGCGAGCAGAAAAAGGCCATCGTGGACCTGCTGTTCAAGACCAA
CCGGAAAGTGACCGTGAAGCAGCTGAAAGAGGACTACTTCAAGAAAATCGAGTGCTTC
GACTCCGTGGAAATCTCCGGCGTGGAAGATCGGTTCAACGCCTCCCTGGGCACATACC
ACGATCTGCTGAAAATTATCAAGGACAAGGACTTCCTGGACAATGAGGAAAACGAGGA
CATTGGTGAAGATATCGTGCTGACCCTGACACTGTTTGAGGACAGAGAGATGATCGAG
GAACGGCTGAAAACCTATGCCCACCTGTTCGACGACAAAGTGATGAAGCAGCTGAAGC
GGCGGAGATACACCGGCTGGGGCAGGCTGAGCCGGAAGCTGATCAACGGCATCCGG
GACAAGCAGTCCGGCAAGACAATCCTGGATTTCCTGAAGTCCGACGGCTTCGCCAACA
GAAACTTCATGCAGCTGATCCACGACGACAGCCTGACCTTTAAAGAGGACATCCAGAA

AGCCCAGGTGTCCGGCCAGGGCGATAGCCTGCACGAGCACATTGCCAATCTGGCCGG
CAGCCCCGCCATTAAGAAGGGCATCCTGCAGACAGTGAAGGTGGTGGACGAGCTCGT
GAAAGTGATGGGCCGGCACAAGCCCGAGAACATCGTGATCGAAATGGCCAGAGAGAA
CCAGACCACCCAGAAGGGACAGAAGAACAGCCGCGAGAGAATGAAGCGGATCGAAGA
GGGCATCAAAGAGCTGGGCAGCCAGATCCTGAAAGAACACCCCGTGGAAAACACCCA
GCTGCAGAACGAGAAGCTGTACCTGTACTACCTGCAGAATGGGCGGGATATGTACGTG
GACCAGGAACTGGACATCAACCGGCTGTCCGACTACGATGTGGACCATATCGTGCCTC
AGAGCTTTCTGAAGGACGACTCCATCGACAACAAGGTGCTGACCAGAAGCGACAAGAA
CCGGGGCAAGAGCGACAACGTGCCCTCCGAAGAGGTCGTGAAGAAGATGAAGAACTA
CTGGCGGCAGCTGCTGAACGCCAAGCTGATTACCCAGAGAAAGTTCGACAATCTGACC
AAGGCCGAGAGAGGCGGCCTGAGCGAACTGGATAAGGCCGGCTTCATCAAGAGACAG
CTGGTGGAAACCCGGCAGATCACAAAGCACGTGGCACAGATCCTGGACTCCCGGATG
AACACTAAGTACGACGAGAATGACAAGCTGATCCGGGAAGTGAAAGTGATCACCCTGA
AGTCCAAGCTGGTGTCCGATTTCCGGAAGGATTTCCAGTTTTACAAAGTGCGCGAGAT
CAACAACTACCACCACGCCCACGACGCCTACCTGAACGCCGTCGTGGGAACCGCCCT
GATCAAAAAGTACCCTAAGCTGGAAAGCGAGTTCGTGTACGGCGACTACAAGGTGTAC
GACGTGCGGAAGATGATCGCCAAGAGCGAGCAGGAAATCGGCAAGGCTACCGCCAAG
TACTTCTTCTACAGCAACATCATGAACTTTTTCAAGACCGAGATTACCCTGGCCAACGG
CGAGATCCGGAAGCGGCCTCTGATCGAGACAAACGGCGAAACCGGGGAGATCGTGTG
GGATAAGGGCCGGGATTTTGCCACCGTGCGGAAAGTGCTGAGCATGCCCCAAGTGAA
TATCGTGAAAAAGACCGAGGTGCAGACAGGCGGCTTCAGCAAAGAGTCTATCCTGCCC
AAGAGGAACAGCGATAAGCTGATCGCCAGAAAGAAGGACTGGGACCCTAAGAAGTAC
GGCGGCTTCGACAGCCCCACCGTGGCCTATTCTGTGCTGGTGGTGGCCAAAGTGGAA
AAGGGCAAGTCCAAGAAACTGAAGAGTGTGAAAGAGCTGCTGGGGATCACCATCATGG
AAAGAAGCAGCTTCGAGAAGAATCCCATCGACTTTCTGGAAGCCAAGGGCTACAAAGA
AGTGAAAAAGGACCTGATCATCAAGCTGCCTAAGTACTCCCTGTTCGAGCTGGAAAAC
GGCCGGAAGAGAATGCTGGCCTCTGCCGGCGAACTGCAGAAGGGAAACGAACTGGCC
CTGCCCTCCAAATATGTGAACTTCCTGTACCTGGCCAGCCACTATGAGAAGCTGAAGG
GCTCCCCCGAGGATAATGAGCAGAAACAGCTGTTTGTGGAACAGCACAAGCACTACCT
GGACGAGATCATCGAGCAGATCAGCGAGTTCTCCAAGAGAGTGATCCTGGCCGACGC
TAATCTGGACAAAGTGCTGTCCGCCTACAACAAGCACCGGGATAAGCCCATCAGAGAG
CAGGCCGAGAATATCATCCACCTGTTTACCCTGACCAATCTGGGAGCCCCTGCCGCCT
TCAAGTACTTTGACACCACCATCGACCGGAAGAGGTACACCAGCACCAAAGAGGTGCT
GGACGCCACCCTGATCCACCAGAGCATCACCGGCCTGTACGAGACACGGATCGACCT
GTCTCAGCTGGGAGGCGACAAAAGGCCGGCGGCCACGAAAAAGGCCGGCCAGGCAA
AAAAGAAAAAGT
