# Supplementary material for: Multifunctional CRISPR-Cas9 with engineered immunosilenced human T cell epitopes
Source: Nat Commun. 2019 Apr 23;10:1842. doi: 10.1038/s41467-019-09693-x (PMC6478683; doi:10.1038/s41467-019-09693-x)
Supplement: Supplementary file 4 — Reporting Summary [file 41467_2019_9693_MOESM4_ESM.pdf]

nature research

Corresponding author(s): SAMIRA KIANI, KAREN S ANDERSON

Life Sciences Reporting Summary

Nature Research wishes to improve the reproducibility of the work that we publish. This form is intended for publication with all accepted life science papers and provides structure for consistency and transparency in reporting. Every life science submission will use this form; some list items might not apply to an individual manuscript, but all fields must be completed for clarity.

For further information on the points included in this form, see Reporting Life Sciences Research. For further information on Nature Research policies, including our data availability policy, see Authors & Referees and the Editorial Policy Checklist.

Please do not complete any field with "not applicable" or n/a. Refer to the help text for what text to use if an item is not relevant to your study. For final submission: please carefully check your responses for accuracy; you will not be able to make changes later.

Experimental design

1. Sample size

Describe how sample size was determined.	Sample sizes for immunological studies and functional analyses were determined based on prior experience and similar publications available in the literature.

2. Data exclusions

Describe any data exclusions.	If PBMCs from a given donor were not reactive to the positive control, the donor was excluded from the study.

3. Replication

Describe the measures taken to verify the reproducibility of the experimental findings.	For ELISpot groups, all donor samples were tested in triplicate and a random selection of donors were tested for validation twice or three times. For functional analysis of Cas9, experiments underwent initial validation in duplicate and were then repeated in triplicate for the final manuscript. ELISA samples were repeated in duplicate.

4. Randomization

Describe how samples/organisms/participants were allocated into experimental groups.	For immunology experiments, participants were ethnically diverse.

5. Blinding

Describe whether the investigators were blinded to group allocation during data collection and/or analysis.	There was no group allocation and investigators were blinded to participants' information.

Note: all in vivo studies must report how sample size was determined and whether blinding and randomization were used.

6. Statistical parameters

For all figures and tables that use statistical methods, confirm that the following items are present in relevant figure legends (or in the Methods section if additional space is needed).

n/a	Confirmed	
☐	☒	The exact sample size (*n*) for each experimental group/condition, given as a discrete number and unit of measurement (animals, litters, cultures, etc.)
☐	☒	A description of how samples were collected, noting whether measurements were taken from distinct samples or whether the same sample was measured repeatedly
☐	☒	A statement indicating how many times each experiment was replicated
☐	☒	The statistical test(s) used and whether they are one- or two-sided<br>*Only common tests should be described solely by name; describe more complex techniques in the Methods section.*
☐	☒	A description of any assumptions or corrections, such as an adjustment for multiple comparisons
☐	☒	Test values indicating whether an effect is present<br>*Provide confidence intervals or give results of significance tests (e.g. P values) as exact values whenever appropriate and with effect sizes noted.*
☐	☒	A clear description of statistics including central tendency (e.g. median, mean) and variation (e.g. standard deviation, interquartile range)
☐	☒	Clearly defined error bars in all relevant figure captions (with explicit mention of central tendency and variation)

See the web collection on statistics for biologists for further resources and guidance.

Software

Policy information about availability of computer code

7. Software

Describe the software used to analyze the data in this study.	FlowJo software was used for the analysis of flow cytometry data related to Cas9 functionality. Attune cytometric software V2.1 was used for the analysis of Cas9 immunogenicity flow cytometry data.

For manuscripts utilizing custom algorithms or software that are central to the paper but not yet described in the published literature, software must be made available to editors and reviewers upon request. We strongly encourage code deposition in a community repository (e.g. GitHub). *Nature Methods* guidance for providing algorithms and software for publication provides further information on this topic.

Materials and reagents

Policy information about availability of materials

8. Materials availability

Indicate whether there are restrictions on availability of unique materials or if these materials are only available for distribution by a third party.	N/A

9. Antibodies

Describe the antibodies used and how they were validated for use in the system under study (i.e. assay and species).	Antibodies used are described in the methods sections. They have all been used in prior publications and verified by their respective companies and come with specification sheets. Antibodies were originally chosen based on their use in published papers with similar applications to our studies. We additionally tested the antibodies on samples known to express the antigen or purified antigen of interest as well as negative controls.

10. Eukaryotic cell lines

a. State the source of each eukaryotic cell line used.	HEK293FT cell lines used in the functional assays were purchased from ATCC.
b. Describe the method of cell line authentication used.	The cell line is used in multiple previous publications and has been validated by the company. The company tests the cell line for DNA rearrangement or unique short tandem repeats to verify cell identity. Additionally we examine cell morphology and periodically use sequencing to verify the identity of the cells.
c. Report whether the cell lines were tested for mycoplasma contamination.	Yes
d. If any of the cell lines used are listed in the database of commonly misidentified cell lines maintained by ICLAC, provide a scientific rationale for their use.	N/A

Animals and human research participants

Policy information about studies involving animals; when reporting animal research, follow the ARRIVE guidelines

11. Description of research animals

Provide all relevant details on animals and/or animal-derived materials used in the study.	N/A

Policy information about studies involving human research participants

12. Description of human research participants

Describe the covariate-relevant population characteristics of the human research participants.	No population characteristics of the healthy donors are available as these are de-identified samples. They were obtained under written informed consent and Arizona State University Institutional Review Board approval.
