# Supplementary material for: Multifunctional CRISPR-Cas9 with engineered immunosilenced human T cell epitopes
Source: Nat Commun. 2019 Apr 23;10:1842. doi: 10.1038/s41467-019-09693-x (PMC6478683; doi:10.1038/s41467-019-09693-x)
Supplement: Supplementary file 5 — Description of Additional Supplementary Files [file 41467_2019_9693_MOESM5_ESM.docx]

**Title:** Supplementary Data 1
**Description:** Predicted T cell epitopes derived from SpCas9 for nonHLA-A*02:01 alleles using the IEDB analysis tool.

**Title:** Supplementary Data 2
**Description:** Predicted MHC class II binding epitopes for the SpCas9 protein to HLA-DRB1 (10 alleles), HLA-DQ (5 alleles), and HLA-DP (8 alleles) using the IEDB analysis tool.
